# Post-COVID Trajectories of Public Primary Healthcare Activity in Greece: Evidence from a 10-Year National Facility-Level Panel (2015–2024)

**DOI:** 10.3390/healthcare14213667

**Published:** 2026-10-28

**Authors:** Margarita Nassi, Sotirios Roussos, Christos Diou, Haralampos Karanikas, Nikolaos Kontodimopoulos

**Affiliations:** 1 Department of Economics and Sustainable Development, Harokopio University, 17676 Athens, Greece; nkontodi@hua.gr; 2 Department of Preventive Medicine, Epidemiology and Medical Statistics, School of Medicine, National and Kapodistrian University of Athens, 11527 Athens, Greece; 3 Department of Informatics and Telematics, Harokopio University, 17778 Athens, Greece; 4 Department of Computer Science and Biomedical Informatics, University of Thessaly, 35131 Lamia, Greece

**Keywords:** primary healthcare, facilities and services utilisation, COVID-19, Greece, longitudinal studies, health services research

## Abstract

**Highlights:**

**What are the main findings?**

• Nationwide longitudinal panel data revealed divergent recovery patterns across different types of primary healthcare visits.
• The analytical approach demonstrates how routinely collected administrative data can support longitudinal monitoring of recorded activity and operational management, even when the number of reporting facilities changes.

**What are the implications of the main findings?**

• The findings identify a persistent difference in recorded activity in scheduled primary healthcare, offering insights to inform health policy, service planning, and resource allocation.
• Distinguishing within-facility change from changes in network composition improves the interpretation of recorded activity patterns and can also help managers to identify persistent changes in recorded activity and may inform service planning.

**Abstract:**

**Background/Objectives**: The Greek public primary healthcare (PHC) system underwent successive structural reforms between 2014 and 2024, while the COVID-19 pandemic profoundly disrupted service delivery. Despite the availability of nationwide administrative data, there is limited longitudinal evidence on how public PHC activity evolved during the post-pandemic period. Using ten years (2015–2024) of routinely collected data from the Greek Ministry of Health’s Business Intelligence system (BI-Health), we examined post-2019 changes in recorded activity among eligible facilities reporting in 2019, alongside annual activity totals and reporting coverage in the evolving public PHC network. **Methods**: We conducted a retrospective observational study of annual facility-level administrative data submitted by public PHC facilities through the BI-Forms subsystem of BI-Health, using the facility-year as the unit of analysis. Facility-level changes were estimated using conditional fixed-effects Poisson panel models with standard errors clustered by facility and 2019 as the reference year. The outcomes represent recorded facility activity rather than population-level utilisation. **Results**: Reporting facilities increased from 461 in 2015 to 653 in 2024. However, changes in recorded activity differed substantially across visit categories. Emergency visits declined sharply during 2020–2021 but approached their 2019 level by 2024 (IRR 0.989, 95% CI 0.927–1.054). Regular visits, however, remained persistently reduced throughout the post-pandemic period (2024 IRR 0.787, 95% CI 0.749–0.826), corresponding to recorded activity approximately one-fifth below 2019 levels in 2024 within the primary regular-visit cohort. The 2024 point estimate for prescription-only visits was modestly below 2019 (IRR 0.944, 95% CI 0.880–1.012). **Conclusions**: These findings suggest that, although recorded emergency activity approached its 2019 level, scheduled public primary-care activity remained lower. Beyond documenting post-pandemic activity patterns, this study demonstrates the value of national longitudinal administrative data for monitoring changes in recorded service activity, with potential applications in service planning. Building on this approach, it also highlights the potential value of a systematic framework to inform decisions in primary healthcare.

## 1. Introduction

Primary healthcare (PHC) serves as a first point of contact for common health needs and supports prevention, chronic disease management, and continuity of care; greater access to PHC is associated with fewer avoidable hospital admissions [1]. In Greece, PHC has repeatedly been identified as the weakest pillar of an otherwise hospital-centred National Health System (ESY), with fragmented provision across public and private providers, high out-of-pocket payments, and the absence of an established gatekeeping mechanism [2,3,4].

The public PHC network was formally reorganised through Law 4238/2014, which established the Primary National Health Network (PEDY), transferred the health units of the National Organization for the Provision of Health Services (EOPYY) to the seven Regional Health Authorities (Health Regions), and redefined EOPYY as a purchasing organisation [5]. A second reform phase, Law 4486/2017, emphasised local accessibility, continuity, prevention and community orientation, and introduced the Local Health Units (TOMY) as a new first-contact structure, especially for vulnerable subpopulations [6,7]. During the early phase of the COVID-19 pandemic, PHC structures were reorganised to support the health-system response [8]. In 2020, the utilisation of essential public healthcare services declined, and excess non-COVID-19 mortality was observed [9]. From 2021, public Health Centres also participated in the national COVID-19 vaccination campaign [10]. Post-pandemic reforms continued with Law 4931/2022 (“Doctor for All”), which introduced the personal doctor scheme, and Law 5157/2024, which also restructured that scheme and established University Health Centres [11,12]. Despite these reforms, public PHC continues to face staffing and equipment shortages [13]. Recent evidence also identifies gaps in care coordination among surveyed primary-care patients aged 45 years and older with chronic conditions [14] and barriers to healthcare access [15].

Successive reforms have sought to strengthen access, continuity and person-centred care in Greek PHC. Assessing progress towards these goals requires regular examination of how public PHC services operate over time. Longitudinal analysis of routinely recorded visits can identify changes in activity that warrant further investigation and can inform the ongoing monitoring of reforms, particularly when changes in the reporting network are considered.

Routinely collected data can support this monitoring, as well as operational management and resource planning. In parallel with European developments towards the secondary use of health data under the European Health Data Space (EHDS) [16], the Greek Ministry of Health operates the Business Intelligence system (BI-Health), through which public healthcare units, including hospitals and public PHC structures, systematically submit operational and administrative information [17]. BI-Health data are openly published and constitute, at present, the only nationwide longitudinal source on the activity of public PHC facilities in Greece. Nevertheless, nationwide analyses of these data across visit categories, facility types and Health Regions remain scarce; most previous work has focused on policy design, the economic crisis, or the acute phase of the pandemic [4,9,18]. Earlier national evidence documented reductions in aggregate public primary-care activity during the first nine months of the pandemic relative to 2017–2019 [9]. The present study extends the observation period through 2024 and combines category-specific within-facility comparisons with an assessment of reporting coverage in the evolving network.

The present study uses ten years of BI-Health administrative extracts (2015–2024) to answer a deliberately specific question: among the public PHC facilities that were recorded in the BI-Health system in 2019, the last pre-pandemic year, how did recorded activity change in 2020–2024 relative to 2019, for regular (scheduled), emergency, and prescription-only visits? Separate analysis of the three visit categories allows divergent trajectories to be identified that could be obscured by changes in total recorded activity. The question is framed in terms of recorded facility activity rather than population utilisation, because the data are facility-level administrative aggregates without information on patients’ residence or on private sector activity. We complement this facility-level analysis with a system-level description of total recorded activity in the evolving public network, a transparent audit of reporting coverage, and robustness analyses.

From a healthcare management perspective, the study addresses a recurrent monitoring problem in expanding service networks: aggregate activity indicators may confound changes in service delivery with changes in the number and composition of reporting facilities. This study therefore proposes an approach to longitudinal monitoring that separates changes in the reporting network from within-facility changes in recorded activity. The distinction provides managers with a more interpretable basis for identifying persistent changes that warrant investigation and monitoring different components of primary care over time. By establishing a harmonised national longitudinal facility panel, the present study also creates a reproducible resource for future research on the organisation and performance of public PHC.

## 2. Materials and Methods

### 2.1. Study Design and Reporting

We conducted a retrospective observational study of routinely collected administrative data, analysed as a facility-year panel. Reporting follows the REporting of studies Conducted using Observational Routinely collected health Data (RECORD) extension of the STROBE statement [19].

### 2.2. Setting and Data Source

The data source is the BI-Forms subsystem of the Greek Ministry of Health’s BI-Health platform, which provides official, publicly available administrative data on public PHC activity [17]. Data are collected by PHC staff and submitted by individual facilities to the Ministry. The available documentation did not allow us to confirm the specific staff roles responsible for data entry, whether reporting was mandatory throughout the study period, or the frequency of submission. The present analysis used annual facility-level totals [17]. The analysed extract includes returns from public PHC facilities across all seven Health Regions for the years 2014–2024 and records, for each facility and year, the number of visits in four administrative categories: regular (scheduled) visits, emergency visits, visits solely for prescription (prescription-only), and a residual category of other visits. Facility type (Health Centre/former PEDY unit, TOMY, regional and local clinics, mental health units, and other structures) was assigned through a static curated lookup table. This lookup did not provide a verified history of organisational changes. The year 2014 was excluded: it was the year of the transfer of former EOPYY units to the Health Regions under Law 4238/2014, with non-comparable reporting procedures [5]. Visit categories were analysed as reported in BI-Forms. Detailed coding rules and any changes to them over time could not be verified from the available extracts.

### 2.3. Study Variables

The three study outcomes were the annual numbers of recorded regular (scheduled), emergency and prescription-only visits per facility. Calendar year was the main explanatory variable in the panel models, with 2019 as the reference year. Facility identifier, facility type and Health Region were used to construct and describe the longitudinal cohorts; facility fixed effects accounted for time-invariant differences between facilities. The primary total was calculated as the sum of the three visit categories for descriptive analyses. The residual “other visits” category was recorded in the source data but was not included in the three study outcomes or the primary total, because its reporting was substantially less complete than that of the other visit categories. The primary total is therefore not an exhaustive measure of recorded PHC activity. Mutual exclusivity of the three principal categories could not be independently verified.

### 2.4. Data Preparation and Quality Control

Facility names were harmonised with a reproducible set of string-cleaning rules before linkage to the facility-type lookup; all unmatched records and name conflicts were exported to quality-control files. A facility identifier was defined as the combination of Health Region and harmonised facility name; a dedicated audit lists names appearing in more than one Health Region. One facility contributed two records for 2015; because these could not be reconciled without arbitrary assumptions, both records were excluded. Numeric fields recorded as text (dashes, dots or blanks) were converted, with such entries treated as missing. Explicitly recorded zeros were retained as zeros; no imputation of missing values was performed.

One facility recorded an implausibly high prescription-only count of 28,132,398 in 2016. The original record was preserved for audit, and only this outcome value was set to missing in the analytical dataset. Its cause could not be verified from the available BI-Health data and portal documentation; less-extreme recording errors may remain undetected.

### 2.5. Analytic Populations

Four analytic populations were defined. (i) The system-level descriptive population comprises all facilities reporting in each year and is used only to describe total recorded activity in the evolving public network. (ii) The primary 2019 baseline cohort is defined separately for each outcome and comprises facilities with a non-missing value of that outcome in 2019, at least one non-missing value in 2020–2024, and at least one positive value in 2019–2024 (the last condition is also required by the fixed-effects estimator). (iii) The strict balanced cohort is the subset of the primary cohort with non-missing values in all years from 2019 to 2024. (iv) The long-balanced cohort is defined separately and requires non-missing values in all years from 2015 to 2024 and at least one positive value during that ten-year period; it supports the assessment of pre-pandemic temporal patterns. For the outlier sensitivity analysis, the long-balanced prescription-only cohort was re-derived with the flagged 2016 value retained, because the outlier lies outside the 2019–2024 window and can therefore only affect the long-balanced panel.

We selected 2019 as the reference year because it was the last pre-pandemic year and had the highest number of facilities observed among the pre-pandemic years (620). Of these, 128 were first observed in the dataset in 2019, including 117 TOMY units. Under our cohort definition, requiring an observed baseline value in 2017 or 2018 would exclude facilities first recorded in 2019 and would therefore define a different reporting population. The choice of 2019 aligns with our objective of describing subsequent changes among facilities recorded in that year. It serves as an observed reference for within-facility comparisons; it does not imply stable pre-pandemic activity or represent a no-pandemic counterfactual.

### 2.6. Statistical Analysis

System-level activity is reported as annual national totals per visit category, calculated by summing non-missing recorded counts, together with the number of facilities reporting each category in each year and an index relative to 2019 (2019 = 100). Because annual totals sum over a changing set of reporting facilities, they describe recorded activity in the observed network and are not used for inference. The analytical strategy was designed to interpret within-facility changes in recorded activity in the context of changes in the composition of the reporting network, thereby supporting longitudinal activity monitoring and operational management.

The primary analysis estimated conditional fixed-effects Poisson regression models using observations from 2019 to 2024, with calendar-year indicators and 2019 as the reference category, separately for each outcome within its 2019 baseline cohort. The conditional Poisson estimator is consistent under correct specification of the conditional mean and standard regularity conditions; facility-clustered robust standard errors allow for overdispersion and within-facility serial correlation, assuming independence across facilities [20]. Facility fixed effects account for time-invariant multiplicative differences in expected recorded activity between facilities. The primary cohorts were unbalanced, and these models do not correct for potentially informative missing reporting or changes in recording practices within facilities. Exponentiated year coefficients are reported as incidence rate ratios (IRRs) with pointwise 95% confidence intervals, interpreted here as common model-based within-facility ratios of expected recorded annual counts relative to 2019. Given the observational design and the documented changes in the reporting system, these IRRs are interpreted as descriptive deviations from the 2019 baseline, not as causal effects of the pandemic.

Robustness analyses re-estimated the models (a) in the strict balanced 2019–2024 cohorts, (b) in the long-balanced 2015–2024 cohorts, and (c) for prescription-only visits, in the long-balanced cohort with the 2016 outlier retained. In the long-balanced panels, joint Wald tests assessed whether the 2015–2018-year coefficients were all zero, corresponding to equality of expected recorded activity in those years with the 2019 level. Regional trajectories are described by Health Region using the primary total (sum of the three main category-specific recorded totals) indexed to 2019; these are descriptive and do not constitute performance rankings. Analyses used Stata 17.0 [21]; two-sided *p*-values below 0.05 were considered statistically significant, without adjustment for multiple comparisons.

To assess changes in reporting coverage, we identified 2019 outcome reporters with unavailable counts in 2024 and calculated their share of the corresponding recorded 2019 visit totals. For prescription-only visits, we additionally compared models in the same 379 facilities with the flagged 2016 value retained or set to missing. Additional outcome-specific analyses estimated log-linear annual changes during 2015–2019 within the long-balanced cohorts and compared 2024 with 2017, 2018 and 2019 within continuously reporting 2017–2024 cohorts, using the same fixed-effects Poisson approach and facility-clustered standard errors.

## 3. Results

### 3.1. Reporting Coverage and Cohort Construction

**Figure 1:**
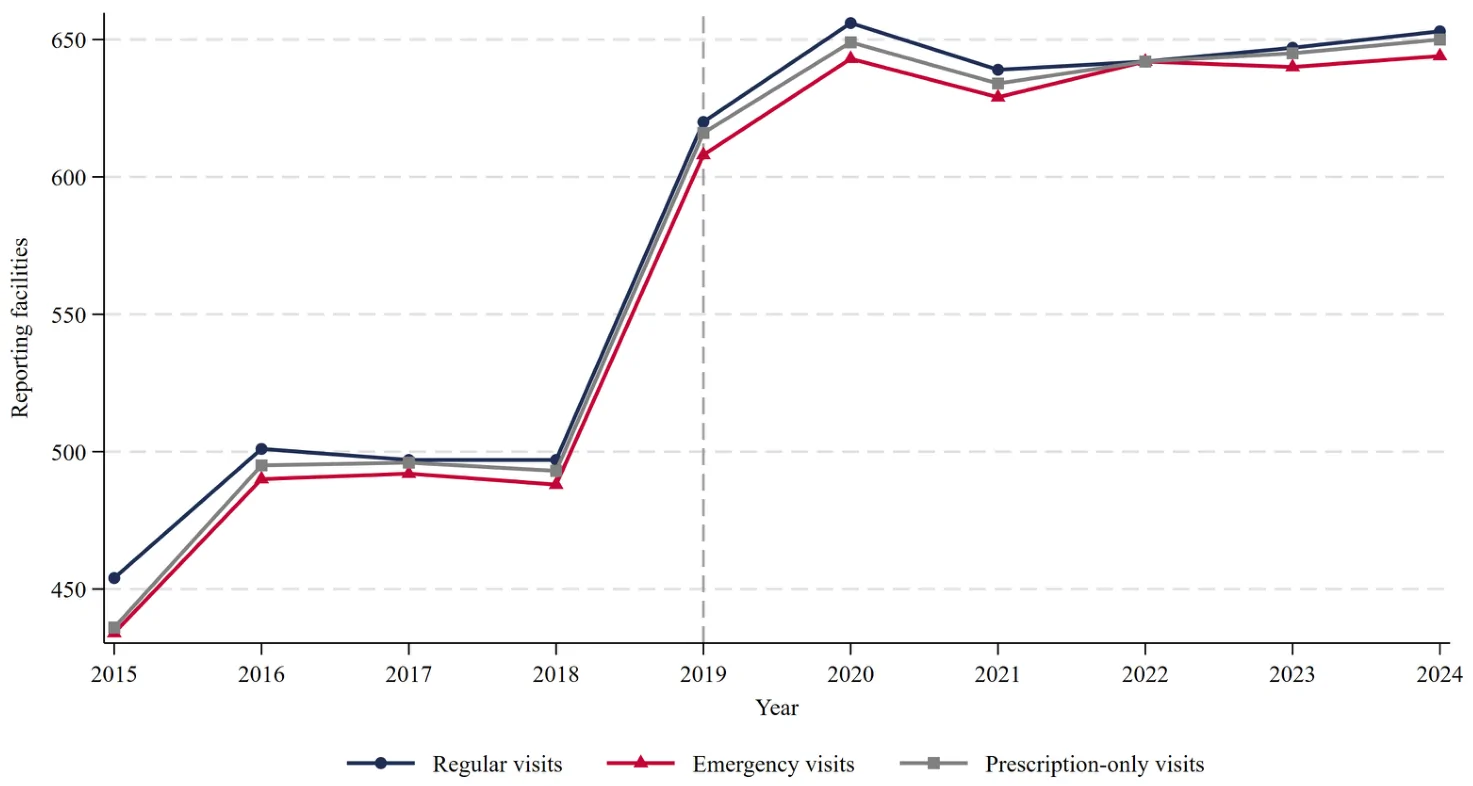
Number of public PHC facilities reporting each visit category to BI-Health, 2015–2024. The dashed line marks 2019.

Table 1 and Figure 1 summarise dataset construction and reporting coverage. The number of reporting facilities rose from 461 in 2015 to 497–510 in 2016–2018, jumped to 620 in 2019 (+24.7% relative to 2018), and stabilised at 639–656 thereafter. Of the 128 facilities first observed in 2019, 117 were TOMY units established under Law 4486/2017, indicating that the 2019 discontinuity reflects the entry of a new facility type into BI-Health reporting rather than a change in activity of existing facilities; this discontinuity motivates the 2019 baseline design. Missing outcome counts within available facility-year records were infrequent for regular, emergency and prescription-only visits (for example, in 2019: 0, 12 and 4 facilities with missing values, respectively) but were substantially more frequent for the residual other-visits category (150 of 461 facilities in 2015), which was therefore not analysed further. Recorded zeros were rare for regular visits (0.8% of reporting facilities in 2019) but more common for emergency (11.7%) and prescription-only visits (7.8%), although the meaning of individual zeros could not be verified.

The primary 2019 baseline cohorts comprised 614 facilities for regular visits (including 305 Health Centres/former PEDY units, 117 TOMY, 87 multipurpose regional clinics, 52 local clinics, 34 mental health units and 19 other structures), 555 for emergency visits and 581 for prescription-only visits; the strict balanced cohorts comprised 572, 516 and 543 facilities, and the long-balanced cohorts 394, 369 and 378 facilities, respectively (Table S1).

Among all 2019 outcome reporters, counts were unavailable in 2024 for 44/620 regular, 47/608 emergency and 44/616 prescription-only reporters. These facilities accounted for 20.0%, 16.3% and 7.2% of the respective recorded 2019 visit volumes; these shares describe baseline activity in facilities with unavailable follow-up counts, not estimated missing visits in 2024.

### 3.2. System-Level Recorded Activity

**Figure 2:**
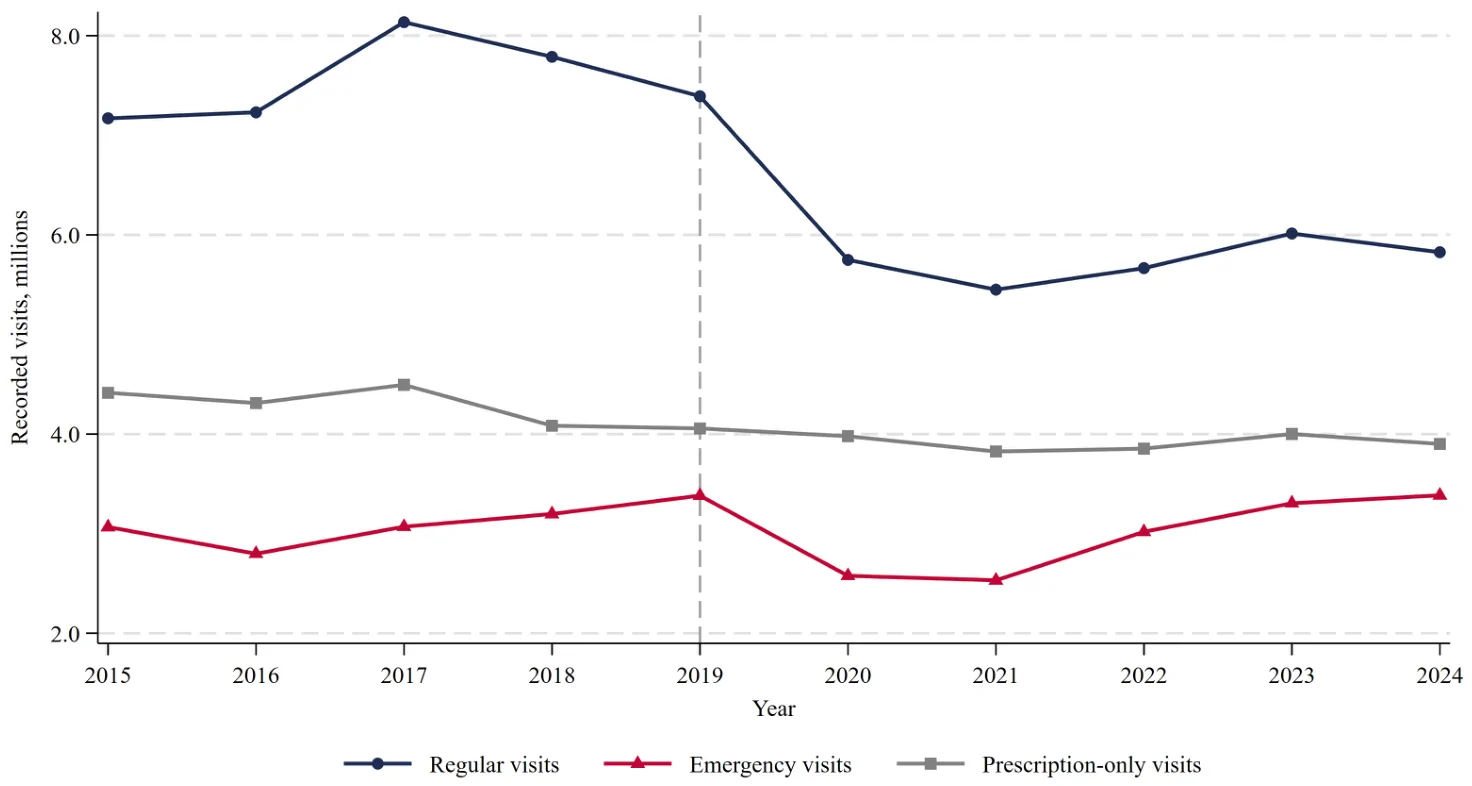
Total recorded visits by category in the public PHC reporting network, 2015–2024 (millions). The dashed line marks 2019. Annual totals include facilities reporting each category in the respective year; reporting coverage varies over time.

Table 2 and Figure 2 show total recorded activity in the observed network. In 2019, public PHC facilities recorded 7.39 million regular, 3.38 million emergency and 4.06 million prescription-only visits (primary total 14.83 million). The primary total fell to 79.6% of the 2019 level in 2021 and recovered only partially to 88.4% by 2024. The three categories diverged sharply: by 2024 emergency visits had returned to 100.1% of 2019 and prescription-only visits to 96.2%, whereas regular visits stood at 78.8%. The 2024 regular-visit total was 21.2% below 2019 despite a larger number of reporters (653 versus 620). A larger reporting count does not exclude a contribution from changes in which facilities reported or from incomplete reporting by facilities with substantial baseline activity.

### 3.3. Within-Facility Change Relative to 2019: Primary Analysis

**Figure 3:**
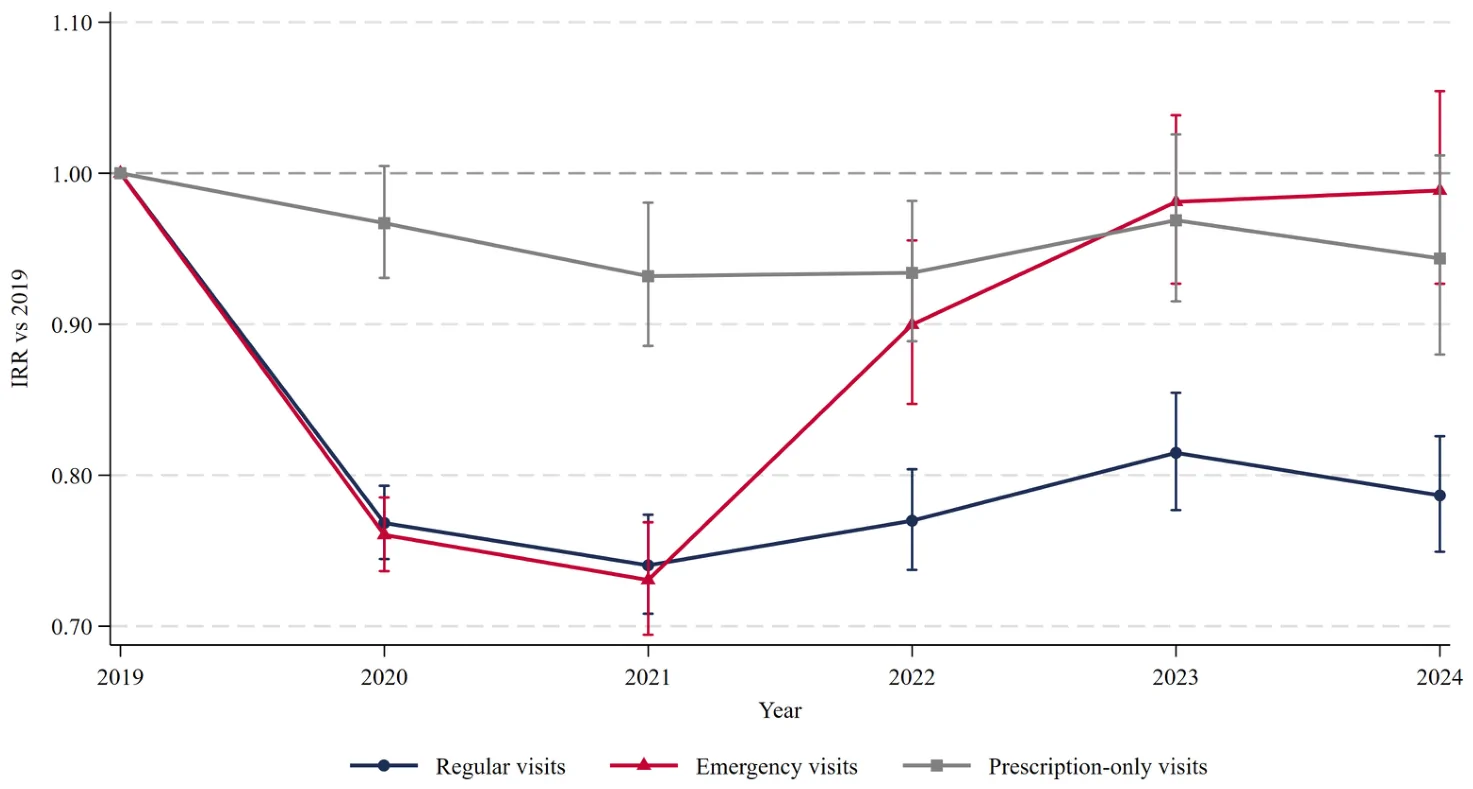
Within-facility deviations of recorded activity from the 2019 level: incidence rate ratios (IRRs) with 95% confidence intervals (CIs) from conditional facility fixed-effects Poisson event-study models, 2019 baseline cohorts.

Table 3 and Figure 3 present the primary within-facility event-study estimates. Regular and emergency visits fell by similar magnitudes at the height of the pandemic: in 2020, IRR 0.768 (95% CI 0.744–0.793) for regular and 0.760 (0.736–0.785) for emergency visits; in 2021, 0.740 (0.708–0.774) and 0.731 (0.694–0.769), respectively. Their subsequent trajectories diverged. Emergency-visit point estimates approached the 2019 reference, with an IRR of 0.900 (0.847–0.955) in 2022, and 0.981 (0.927–1.038) in 2023 and 0.989 (0.927–1.054) in 2024. Regular visits remained below their 2019 level: the IRR was 0.770 (0.737–0.804) in 2022, 0.815 (0.777–0.855) in 2023 and 0.787 (0.749–0.826) in 2024, corresponding to a persistent deficit of 18.5–23.0% relative to 2019 in each year from 2022 to 2024 (all *p* < 0.001). Prescription-only visits declined modestly, with IRRs between 0.932 (2021) and 0.969 (2023) and a 2024 estimate of 0.944 (0.880–1.012).

### 3.4. Robustness and Pre-Trend Analyses

The pattern was essentially unchanged across all robustness analyses (Table 4). In the strict balanced panels, the 2024 IRRs were 0.791 (0.754–0.831) for regular and 0.978 (0.917–1.043) for emergency visits; in the long-balanced panels, 0.763 (0.723–0.805) and 0.972 (0.907–1.040), respectively. In an additional sensitivity analysis using the same 379 facilities, setting the flagged 2016 prescription-only value to missing reduced the 2016/2019 IRR from 9.88 to 1.17. The 2024/2019 IRR was unchanged at 0.93 (95% CI 0.86–1.01).

Joint Wald tests rejected equality of the 2015–2018 annual levels with the 2019 level in all three long-balanced cohorts (regular: χ^2^(4) = 112.5; emergency: χ^2^(4) = 32.6; prescription-only: χ^2^(4) = 55.9; all *p* < 0.001; Table S5). These are tests of annual level contrasts, not linear trends; the full year-specific estimates are presented in Table S3. Separate log-linear models fitted over 2015–2019 yielded annual count ratios of 0.992 (95% CI 0.975–1.009) for regular visits, 1.024 (1.005–1.043) for emergency visits and 0.955 (0.934–0.977) for prescription-only visits. These estimates describe pre-period temporal patterns and do not provide a no-pandemic counterfactual.

### 3.5. Regional Descriptive Trajectories

**Figure 4:**
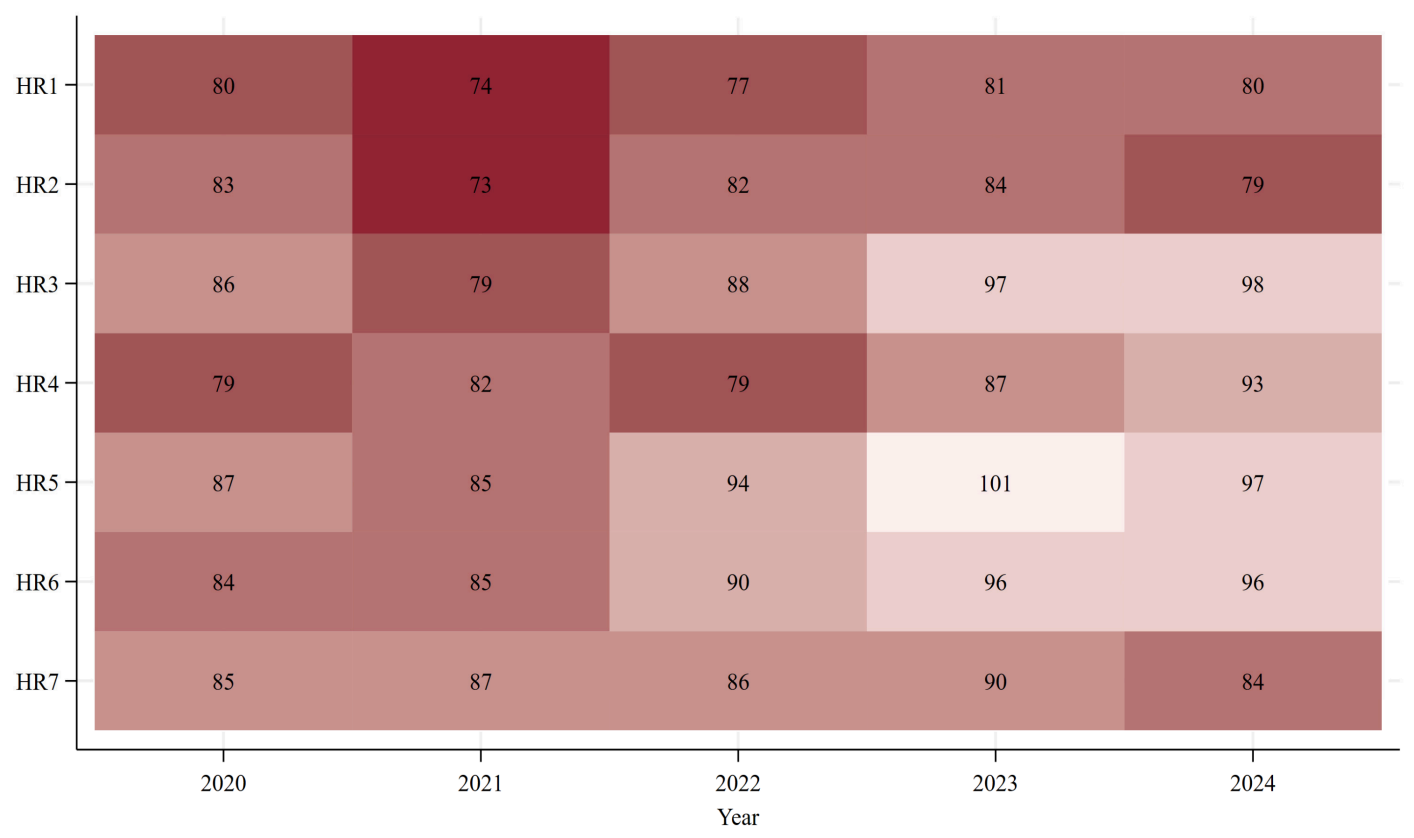
Recorded primary-total activity by Health Region, indexed to 2019 (2019 = 100), 2020–2024. Primary total is the sum of regular, emergency and prescription-only visits. Darker shading indicates a larger deficit relative to 2019. Values describe unadjusted changes in each region’s evolving reporting network and are not performance rankings.

Regional descriptive trajectories of the primary total (Figure 4, Table S2) show the 2021 trough ranging from 72.9% of the 2019 level in the 2nd Health Region to 86.9% in the 7th. In 2024, the recorded primary-total index was highest in the 3rd (98.4), 5th (96.9) and 6th (95.8) Health Regions, and lowest in the 2nd (78.7), 1st (80.2) and 7th (84.3). These figures describe recorded activity in each region’s evolving facility network and should not be read as performance rankings.

## 4. Discussion

### 4.1. Interpretation of the Results

This nationwide facility-level panel study of ten years of Greek administrative data yields one central finding: after parallel declines of about one-quarter in 2020–2021, recorded emergency activity in public PHC facilities returned approximately to its 2019 level by 2023–2024, whereas recorded scheduled (regular) activity recovered only partially, remaining below its 2019 level in every year from 2020 through 2024 within the same facilities. The persistence, magnitude and robustness of this asymmetry—across strictly balanced panels, a decade-long balanced panel, and outlier handling—are consistent with a sustained difference in recorded scheduled public primary-care activity that is not explained solely by changes in panel composition. From a health-system management perspective, these findings show that aggregate service volumes alone are insufficient to assess changes in recorded activity, because different components of primary care may follow markedly different operational trajectories.

The system-level figures reinforce this interpretation. Total recorded regular visits in 2024 were 21% below 2019 even though the reporting network was larger (653 versus 620 facilities), including the TOMY units whose entry into the BI-Health system in 2019 we document explicitly. In other words, a larger reporting network recorded substantially less scheduled activity than in 2019. Because the set of reporting facilities changed, this system-level comparison should be read alongside the within-facility estimates.

Studies reported disruptions in primary care and other essential healthcare service utilisation during the early stages of the COVID-19 pandemic, with the extent of the reduction varying across healthcare systems and types of services [22,23]. In Poland, publicly financed PHC outpatient physician consultations, including teleconsultations, remained below their pre-pandemic volume: in 2021, they amounted to approximately 75% of the 2019 total [23]. In England, overall general-practice consultation rates were higher in 2021–2022 than before the pandemic, despite fewer face-to-face consultations [24]. In seven Danish general practices, annual patient contacts had returned to their pre-pandemic level by 2022–2023, although face-to-face contacts remained fewer [25]. Although recorded scheduled visits in Greece remained below their 2019 level through 2024, the differing study periods, healthcare systems, and contact definitions mean that direct comparisons with these studies are not warranted.

Several non-exclusive mechanisms could produce this pattern, and our data cannot discriminate among them. On the supply side, documented shortages and attrition of PHC personnel may have constrained bookable capacity, while changes in facility opening hours or the redeployment of staff to vaccination and pandemic-related tasks may also have contributed to changes in recorded visit counts. Future studies integrating routinely collected administrative data with workforce and organisational information may help disentangle the relative contribution of these mechanisms. On the demand side, care may have shifted to the private sector, to hospital outpatient departments, or to remote channels: the expansion of e-prescription and telephone consultation plausibly may have substituted for some visit-based contacts, and the small persistent deficit in prescription-only visits (≈5% in 2024) may be consistent with prescription renewals migrating to electronic channels rather than disappearing. The personal doctor scheme introduced in 2022 [11] may also have redirected some first-contact activity outside the structures captured by BI-Forms. More broadly, such shifts in care delivery may have unequal implications across population groups; evidence from the United States suggests that the pandemic compounded pre-existing barriers to healthcare among immigrants [26]. Distinguishing these mechanisms requires linked patient-level or claims data.

In the outcome-specific balanced 2017–2024 cohorts, recorded regular and prescription-only visit counts were lower in 2019 than in 2017 and 2018. The 2024 point estimates were below all three pre-pandemic reference levels, with larger estimated shortfalls relative to 2017 or 2018 than relative to 2019, holding facility composition constant. Thus, the direction of the point estimates was consistent across the reference years examined, whereas the magnitude of the gap depended on the reference. These comparisons describe changes in recorded facility activity and do not isolate the contribution of the pandemic from pre-existing changes or other contemporaneous developments.

### 4.2. Strengths and Limitations

Strengths of this study include nationwide coverage of public PHC facilities recorded in the BI-Health system; a design that separates within-facility change from compositional change in the reporting network—a distinction that matters greatly here, since the number of facilities reporting to BI-Health increased by 24.7% between 2018 and 2019; outcome-specific cohorts that avoid imputation; robustness analyses; and reporting according to RECORD [19]. Beyond the present analyses, the longitudinal facility panel may provide a starting point for future predictive analytics and operational decision-support research. Such applications would require further data-quality assessment, additional relevant variables, and separate development, validation and impact evaluation. The panel therefore represents not only a research dataset but also a reusable analytical asset for routine monitoring of recorded activity within the public PHC system.

This study has several limitations related to the administrative data and the assumptions of the analytical approach. First, the data consist of annual visit counts by facility rather than patient-level measures of population utilisation or need. They do not identify individual patients or describe the size and characteristics of the populations served by each facility; activity outside the public PHC network is also not captured. We therefore cannot examine differences between population subgroups (i.e., migrant or refugee status) or determine how local population changes contributed to the observed trends, and we avoid per capita rates and regional performance comparisons. Second, reporting coverage and, potentially, recording practices changed over time, most notably in 2019. Although the analytical design explicitly separates within-facility change from changes in the composition of the reporting network, changes in recording behaviour within individual facilities may have occurred and cannot be completely excluded. Facilities without the observations required for each analytic cohort were excluded; if their activity differed systematically from that of included facilities, the within-facility results may not represent all public PHC facilities. Similarly, entry into or exit from BI-Health reporting cannot by itself establish that a facility opened or closed. Annual facility-level information on staffing, opening hours, service capacity and services offered was not available in the analytical dataset; therefore, the contribution of changes in these factors to recorded visit counts could not be assessed. Third, some aspects of data quality remain uncertain. The interpretation of recorded zeros is partly ambiguous (true zero activity, non-provision of a service or non-reporting). Retaining explicit zeros and defining outcome-specific cohorts do not resolve this ambiguity or eliminate selection bias. In addition, the residual other-visits category was of insufficient data quality to support reliable analysis and was therefore excluded from the inferential analyses. Fourth, facilities were identified by harmonised names within Health Regions, and regional audits supported quality control but did not establish organisational continuity across mergers, splits, reclassification, or changes in identifiers. Such changes may have affected longitudinal linkage and cohort membership; their impact could not be quantified from the available extracts. Fifth, the fixed-effects Poisson models assume a correctly specified multiplicative conditional mean. Facility-clustered standard errors account for dependence within facilities but not for dependence between facilities. Finally, annual aggregation precludes analysis of within-year dynamics, including individual lockdown periods. Importantly, these limitations also identify clear priorities for future research. Future research should integrate activity records with workforce, organisational and population information to investigate the mechanisms underlying the observed patterns. Future assessments of PHC access should also consider migrants, refugees and other vulnerable groups, using appropriate patient-level information and population denominators.

The findings have direct policy relevance. The near return of recorded emergency visits to their 2019 level alongside a persistent one-fifth shortfall in recorded scheduled activity warrants further investigation in the context of policy efforts to strengthen planned, continuous primary care [6,11]. The data do not establish whether this shortfall reflects capacity, population need, use of other providers or changes in recording. Assessing these possibilities would help determine whether, and where, additional support for scheduled care is needed. Our results also demonstrate both the value and the current limits of the platform BI-Health as a monitoring infrastructure: it enabled a national ten-year facility panel, but its usefulness would increase substantially with stable facility identifiers, documented reporting standards, and eventual linkage capability consistent with the EHDS agenda [16]. Further development of platforms such as BI-Health could enable routinely collected data to support not only retrospective reporting but also continuous organisational learning and evidence-informed management. Learning health systems seek to create repeated cycles in which data generated during routine service delivery are analysed, translated into knowledge and subsequently used to adapt practice, allocate resources and improve outcomes [27,28,29]. Achieving this potential requires more than increasing the volume of data collected. Stable facility identifiers, common data definitions, complete and timely reporting, automated quality controls, transparent metadata, interoperability and appropriate governance are necessary to produce findings that are sufficiently valid, comparable and actionable for management and policy decisions. Health-system data can strengthen the applicability and implementation of scientific evidence, but their limitations, provenance and quality must remain transparent [30].

For managers of public PHC systems, monitoring recorded visits separately by category, facility and Health Region can help identify persistent changes that warrant closer investigation. Before considering changes to staffing or service provision, managers would need to examine possible explanations, including reporting practices, available capacity and activity outside the observed public PHC network. Visit counts can indicate where further investigation is needed but cannot establish the causes of the observed changes on their own. These digital improvements are becoming particularly important in the context of the EHDS, which establishes a common European legal and technical framework for the secure access, exchange and reuse of electronic health data for healthcare delivery, research, innovation, public health and policymaking [16,31]. Investment in data collection should therefore be regarded not merely as an information-technology expenditure, but as a core health-system capacity that supports operational management, service planning, research and accountability. Higher-quality and interoperable data would allow administrative platforms such as BI-Health to evolve from repositories of retrospectively reported activity into infrastructures capable of supporting continuous performance monitoring, potential cross-provider comparisons, evaluation of organisational reforms and evidence-informed allocation of resources, when the additional data needed for those purposes are available. At the same time, the development of such infrastructures must remain patient-centred, ensuring transparency, meaningful patient participation, data protection and equitable access to the benefits of digital transformation [28,31].

Patient-centred data architectures may facilitate the creation of longitudinal records that support decisions at the point of care while also generating higher-quality datasets for research, planning and policymaking [32]. At the same time, meaningful citizen and patient involvement, transparency, privacy protection and public trust will be essential if wider data reuse is to retain social legitimacy and contribute equitably to health-system improvement [33].

From a management science perspective, the principal contribution of this study extends beyond describing post-pandemic patterns of recorded activity. By separating within-facility changes in recorded activity from changes in the composition of the reporting network, the analysis demonstrates how routinely collected administrative data can be transformed into management-relevant evidence. This longitudinal analytical approach, if applied routinely to timely reported data, can help managers monitor recorded activity, identify persistent changes early and prioritise questions for further investigation.

Beyond its role as a retrospective monitoring infrastructure, BI-Health has the potential to evolve into a platform for operational intelligence in public PHC, consistent with the broader transition towards learning health systems that use routinely collected data to support continuous organisational learning and operational decision-making [28]. By establishing a harmonised longitudinal facility-level panel, the present study creates the conditions for future predictive and decision-support models capable of identifying facilities at risk of sustained service disruption, anticipating service demand and supporting evidence-informed management decisions. Developing and validating such models would require additional data beyond the visit counts analysed in this article. Such approaches would not replace conventional statistical inference, which remains essential for transparent evaluation of health-system performance, but would complement it by enabling prospective, facility-level operational support.

## 5. Conclusions

Among the Greek public PHC facilities present in the national BI-Health system in 2019, recorded emergency activity approached its 2019 level by 2023–2024, whereas scheduled activity remained approximately 20% below its 2019 level through 2024, without returning to the 2019 level and with results that were robust across all sensitivity analyses. This divergent trajectory indicates that changes in aggregate recorded activity should not automatically be interpreted as restoration and/or recovery of all components of service delivery. In particular, the recovery of emergency contacts alongside the persistent contraction of scheduled activity warrants further investigation into the implications of scheduled service provision, including its relationship with staffing, service capacity and care delivered outside the observed network. Monitoring recorded scheduled activity should therefore form part of the assessment of the Greek PHC reforms. These comparisons describe recorded facility activity and do not establish a causal pandemic effect or changes in population access.

The study also demonstrates the analytical value of distinguishing changes occurring within existing facilities from changes arising through the expansion or altered composition of the reporting network. National totals may obscure persistent differences in recorded activity when facilities enter or leave an administrative system over time. The present approach provides a more interpretable assessment of longitudinal changes in recorded activity by combining system-level activity measures, transparent reporting and coverage audits, facility fixed-effects models, and balanced-panel sensitivity analyses.

Thus, this study provides evidence of the need for a systematic framework to inform decisions in PHC. Such a framework would use stable facility identifiers, consistent visit definitions and routine checks of reporting completeness to track the recorded activity by facility and/or service category. It would also distinguish changes within facilities from changes in the reporting network, helping PHC managers identify patterns that warrant investigation and whether further actions are needed. Data on staffing, the characteristics of the population in the area served by each facility and other providers would be needed to assess the causes of these patterns and their implications for access to primary care. Health data platforms and analytical methods can generate evidence to inform the development of more efficient, responsive and patient-centred health systems. Any predictive models or decision-support tools developed using these data would require validation and evaluation of their effects on care delivery and patient outcomes before routine implementation.

## Figures and Tables

**Table 1:** Reporting coverage and dataset construction by year, BI-Health public PHC extract, 2015–2024.

Year	Facilities Observed	Facilities Reporting a Non-Missing Outcome Count				Facilities First Observed
		Regular	Emergency	Prescription-Only	Other	
2015	461	454	434	436	311	461
2016	510	501	490	495	452	60
2017	510	497	492	496	473	0
2018	497	497	488	493	465	6
2019	620	620	608	616	573	128
2020	656	656	643	649	602	35
2021	639	639	629	634	597	25
2022	642	642	642	642	642	18
2023	647	647	640	645	606	11
2024	653	653	644	650	606	2

***Notes:*** *PHC, primary healthcare; BI-Health, Business Intelligence system of the Greek Ministry of Health. Values are numbers of facilities after data cleaning. Each observed facility contributes one facility-year record per year; therefore, the number of records equals the number of facilities observed. Reporting denotes a non-missing outcome count, including recorded zeros. First observed denotes the first appearance in the 2015–2024 extract, not necessarily the opening of a facility. The 461 facilities in 2015 represent the initial observed set. Two unreconciled records for one facility in 2015 were excluded.*

**Table 2:** Recorded public PHC visit counts and indices relative to 2019, 2015–2024.

Year	Regular Visits		Emergency Visits		Prescription-Only Visits		Primary Total	
	Count	Index	Count	Index	Count	Index	Count	Index
2015	7,169,883	97.0	3,066,172	90.7	4,414,558	108.8	14,650,613	98.8
2016	7,230,638	97.8	2,797,929	82.7	4,311,104	106.3	14,339,671	96.7
2017	8,136,056	110.1	3,069,329	90.8	4,494,354	110.8	15,699,739	105.9
2018	7,787,658	105.4	3,197,076	94.5	4,083,772	100.7	15,068,506	101.6
**2019**	**7,391,962**	**100.0**	**3,381,554**	**100.0**	**4,056,122**	**100.0**	**14,829,638**	**100.0**
2020	5,748,785	77.8	2,576,685	76.2	3,978,369	98.1	12,303,839	83.0
2021	5,450,209	73.7	2,530,733	74.8	3,824,627	94.3	11,805,569	79.6
2022	5,665,670	76.6	3,018,020	89.2	3,853,392	95.0	12,537,082	84.5
2023	6,013,527	81.4	3,305,355	97.7	4,000,571	98.6	13,319,453	89.8
2024	5,825,468	78.8	3,384,010	100.1	3,900,738	96.2	13,110,216	88.4

***Notes:*** *PHC, primary healthcare. Annual totals sum available recorded counts for each visit category; the reporting facilities vary by year and category (Table 1). Indices are calculated as the annual count divided by the corresponding 2019 count, multiplied by 100. The bold row identifies the 2019 reference year (index = 100). Primary total is the sum of regular, emergency and prescription-only visit totals and excludes other visits. These figures describe recorded activity in the changing reporting network, not within-facility changes or population-level utilisation.*

**Table 3:** Primary 2019 baseline cohort event-study estimates: incidence rate ratios versus 2019.

Outcome	Year	IRR (95% CI)	*p* Value	Difference vs. 2019 (%)
Regular visits (614 facilities; 3537 observations)	2019	1.000 (reference)	—	0.0
	2020	0.768 (0.744–0.793)	<0.001	−23.2
	2021	0.740 (0.708–0.774)	<0.001	−26.0
	2022	0.770 (0.737–0.804)	<0.001	−23.0
	2023	0.815 (0.777–0.855)	<0.001	−18.5
	2024	0.787 (0.749–0.826)	<0.001	−21.3
Emergency visits (555 facilities; 3196 observations)	2019	1.000 (reference)	—	0.0
	2020	0.760 (0.736–0.785)	<0.001	−24.0
	2021	0.731 (0.694–0.769)	<0.001	−26.9
	2022	0.900 (0.847–0.955)	<0.001	−10.0
	2023	0.981 (0.927–1.038)	0.509	−1.9
	2024	0.989 (0.927–1.054)	0.726	−1.1
Prescription-only visits (581 facilities; 3359 observations)	2019	1.000 (reference)	—	0.0
	2020	0.967 (0.931–1.005)	0.086	−3.3
	2021	0.932 (0.886–0.980)	0.007	−6.8
	2022	0.934 (0.889–0.982)	0.007	−6.6
	2023	0.969 (0.915–1.026)	0.276	−3.1
	2024	0.944 (0.880–1.012)	0.103	−5.6

***Notes:*** *IRR, incidence rate ratio; CI, confidence interval. Estimates are from separate conditional facility fixed-effects Poisson models with standard errors clustered by facility. The 2019 reference IRR is fixed at 1.000; no CI or p value is estimated for this category. IRRs describe ratios of expected recorded annual counts relative to 2019 within facilities. Percentage differences equal (IRR − 1) × 100 and are calculated from unrounded estimates. CIs are pointwise, and no adjustment was made for multiple comparisons.*

**Table 4:** Robustness analyses: IRR (95% CI) versus 2019 in 2020, 2021 and 2024.

Year	Regular Visits	Emergency Visits	Prescription-Only Visits
**A. Primary 2019 baseline cohort**			
Facilities, N	614	555	581
2019	1.000 (reference)	1.000 (reference)	1.000 (reference)
2020	0.768 (0.744–0.793)	0.760 (0.736–0.785)	0.967 (0.931–1.005)
2021	0.740 (0.708–0.774)	0.731 (0.694–0.769)	0.932 (0.886–0.980)
2024	0.787 (0.749–0.826)	0.989 (0.927–1.054)	0.944 (0.880–1.012)
**B. Strict balanced panel 2019–2024**			
Facilities, N	572	516	543
2019	1.000 (reference)	1.000 (reference)	1.000 (reference)
2020	0.779 (0.756–0.802)	0.740 (0.714–0.766)	0.978 (0.941–1.017)
2021	0.746 (0.712–0.781)	0.723 (0.686–0.763)	0.943 (0.895–0.993)
2024	0.791 (0.754–0.831)	0.978 (0.917–1.043)	0.951 (0.887–1.021)
**C. Long-balanced panel 2015–2024**			
Facilities, N	394	369	378
2019	1.000 (reference)	1.000 (reference)	1.000 (reference)
2020	0.752 (0.727–0.777)	0.731 (0.705–0.759)	0.955 (0.916–0.995)
2021	0.723 (0.687–0.762)	0.722 (0.683–0.762)	0.921 (0.868–0.976)
2024	0.763 (0.723–0.805)	0.972 (0.907–1.040)	0.927 (0.855–1.005)
**D. Outlier retained—long-balanced panel**			
Facilities, N	—	—	379
2019	—	—	1.000 (reference)
2020	—	—	0.950 (0.911–0.991)
2021	—	—	0.919 (0.868–0.974)
2024	—	—	0.929 (0.857–1.006)

***Notes:*** *IRR, incidence rate ratio; CI, confidence interval; and N, number of facilities. Estimates are from conditional facility fixed-effects Poisson models with facility-clustered standard errors. The 2019 IRR is fixed at 1.000 (reference); no CI is estimated. —, not applicable. The outlier-retained analysis concerns only prescription-only visits, with the flagged 2016 value retained and the long-balanced cohort re-derived. A comparison using the same 379 facilities with that value retained or set to missing is reported in Section 3.4.*

## Data Availability

The study was based on publicly available, aggregated administrative data obtained from the BI-Forms subsystem of the BI-Health platform of the Greek Ministry of Health. The data were extracted, cleaned, harmonised and restructured by the authors for the purposes of the present analysis. The original data are available from the Greek Ministry of Health at https://www.moh.gov.gr/articles/bihealth/311-bi-health-eisagwgh-stoixeiwn-monadwn-ygeias (accessed on 7 October 2025). The data used in this study were downloaded on 7 October 2025 at 17:55 Greek local time. The analyses reflect that extract; subsequent revisions to the publicly available records could not be verified.

## Supplementary Materials

The following supporting information can be downloaded at: https://www.mdpi.com/article/10.3390/healthcare14213667/s1, Table S1. Composition of the outcome-specific cohorts by Health Region and facility type; Table S2. Annual recorded activity and reporting coverage by Health Region, 2015–2024; Table S3. Full year-specific estimates in the long-balanced 2015–2024 cohorts; Table S4. Long-balanced prescription-only sensitivity retaining the extreme 2016 value; Table S5. Joint Wald tests of the 2015–2018 annual level contrasts relative to 2019.

## Abbreviations

The following abbreviations are used in this manuscript:

**Abbreviation**	**Definition**
BI-Forms	Forms subsystem of the BI-Health platform
BI-Health	Business Intelligence system of the Greek Ministry of Health
CI	Confidence interval
COVID-19	Coronavirus disease 2019
EHDS	European Health Data Space
EOPYY	National Organization for the Provision of Health Services
ESY	National Health System
IRR	Incidence rate ratio
PEDY	Primary National Health Network
PHC	Primary healthcare
RECORD	REporting of studies Conducted using Observational Routinely collected health Data
STROBE	Strengthening the Reporting of Observational Studies in Epidemiology
TOMY	Local Health Units
