# Supplementary material for: Post-COVID Trajectories of Public Primary Healthcare Activity in Greece: Evidence from a 10-Year National Facility-Level Panel (2015–2024)
Source: Healthcare (Basel). 2026 Oct 28;14(21):3667. doi: 10.3390/healthcare14213667 (PMC13763244; doi:10.3390/healthcare14213667)
Supplement: Supplementary file 1 [file healthcare-14-03667-s001.zip › healthcare-4575865-supplementary.pdf]

Supplementary material

Post-COVID trajectories of public primary health care activity in Greece: evidence from a 10-year national facility-level panel (2015–2024)

Table S1. Composition of the outcome-specific cohorts by Health Region and facility type

Regular visits

Health Region	Facility type	Primary 2019 cohort	Strict balanced 2019–2024	Long balanced 2015–2024
HR1	HC-HU	30	8	8
HR1	Mental HU	0	0	0
HR1	Other	4	3	2
HR1	Local Clinics	19	18	18
HR1	TOMY	12	11	0
HR2	HC-HU	49	48	48
HR2	Mental HU	6	6	0
HR2	Other	5	3	3
HR2	PPI	37	36	34
HR2	Local Clinics	8	6	6
HR2	TOMY	20	20	0
HR3	HC-HU	31	31	31
HR3	Mental HU	4	4	0
HR3	Other	4	2	1
HR3	PPI	4	4	3
HR3	Local Clinics	12	10	9
HR3	TOMY	10	10	0
HR4	HC-HU	45	45	45
HR4	Mental HU	6	5	0
HR4	Other	0	0	0
HR4	PPI	9	9	5
HR4	Local Clinics	10	6	4
HR4	TOMY	14	14	0
HR5	HC-HU	43	43	40

Health Region	Facility type	Primary 2019 cohort	Strict balanced 2019–2024	Long balanced 2015–2024
HR5	Mental HU	3	3	0
HR5	Other	5	5	1
HR5	PPI	14	14	12
HR5	Local Clinics	1	1	0
HR5	TOMY	16	16	0
HR6	Unknown	0	0	0
HR6	HC-HU	89	89	86
HR6	Mental HU	11	11	0
HR6	Other	1	1	0
HR6	PPI	19	18	18
HR6	Local Clinics	1	1	1
HR6	TOMY	33	33	0
HR7	HC-HU	18	18	16
HR7	Mental HU	4	4	0
HR7	Other	0	0	0
HR7	PPI	4	3	2
HR7	Local Clinics	1	1	1
HR7	TOMY	12	12	0
All regions	All types	614	572	394

Note: HC–HU combines Health Centres and Health Units for presentation; their administrative identifiers remain separate. Mental HU: mental health units; PPI: multipurpose regional clinics; TOMY: local health units. The strict balanced cohort is a subset of the primary cohort. The long-balanced cohort requires a non-missing count in all ten years and at least one positive count during 2015–2024. The flagged 2016 prescription-only value is excluded before eligibility is determined.

Table S1. Composition of the outcome-specific cohorts by Health Region and facility type (continued)

Emergency visits

Health Region	Facility type	Primary 2019 cohort	Strict balanced 2019–2024	Long balanced 2015–2024
HR1	HC-HU	30	8	8
HR1	Mental HU	0	0	0
HR1	Other	4	3	1
HR1	Local Clinics	19	18	18
HR1	TOMY	12	11	0
HR2	HC-HU	49	48	46
HR2	Mental HU	2	2	0
HR2	Other	5	3	3
HR2	PPI	37	36	33
HR2	Local Clinics	8	6	5
HR2	TOMY	20	18	0
HR3	HC-HU	21	21	24
HR3	Mental HU	0	0	0
HR3	Other	1	1	0
HR3	PPI	1	1	1
HR3	Local Clinics	1	1	2
HR3	TOMY	6	6	0
HR4	HC-HU	44	44	45
HR4	Mental HU	2	1	0
HR4	Other	0	0	0
HR4	PPI	9	9	2
HR4	Local Clinics	9	6	5
HR4	TOMY	14	14	0
HR5	HC-HU	43	43	39
HR5	Mental HU	2	2	0
HR5	Other	5	5	1

Health Region	Facility type	Primary 2019 cohort	Strict balanced 2019–2024	Long balanced 2015–2024
HR5	PPI	10	10	12
HR5	Local Clinics	1	1	0
HR5	TOMY	16	16	0
HR6	Unknown	0	0	0
HR6	HC-HU	89	89	88
HR6	Mental HU	4	4	0
HR6	Other	1	1	0
HR6	PPI	19	18	17
HR6	Local Clinics	1	1	1
HR6	TOMY	33	33	0
HR7	HC-HU	18	18	17
HR7	Mental HU	2	2	0
HR7	Other	0	0	0
HR7	PPI	4	3	0
HR7	Local Clinics	1	1	1
HR7	TOMY	12	12	0
All regions	All types	555	516	369

Note: HC–HU combines Health Centres and Health Units for presentation; their administrative identifiers remain separate. Mental HU: mental health units; PPI: multipurpose regional clinics; TOMY: local health units. The strict balanced cohort is a subset of the primary cohort. The long-balanced cohort requires a non-missing count in all ten years and at least one positive count during 2015–2024. The flagged 2016 prescription-only value is excluded before eligibility is determined.

Table S1. Composition of the outcome-specific cohorts by Health Region and facility type (continued)

Prescription-only visits

Health Region	Facility type	Primary 2019 cohort	Strict balanced 2019–2024	Long balanced 2015–2024
HR1	HC-HU	24	7	7
HR1	Mental HU	0	0	0
HR1	Other	2	1	0
HR1	Local Clinics	18	15	13
HR1	TOMY	12	11	0
HR2	HC-HU	47	46	44
HR2	Mental HU	6	6	0
HR2	Other	5	3	3
HR2	PPI	37	36	34
HR2	Local Clinics	5	4	4
HR2	TOMY	20	20	0
HR3	HC-HU	30	30	30
HR3	Mental HU	3	3	0
HR3	Other	4	2	1
HR3	PPI	4	4	3
HR3	Local Clinics	4	2	9
HR3	TOMY	10	10	0
HR4	HC-HU	45	45	42
HR4	Mental HU	5	4	0
HR4	Other	0	0	0
HR4	PPI	9	9	5
HR4	Local Clinics	10	6	5
HR4	TOMY	14	14	0
HR5	HC-HU	43	43	40
HR5	Mental HU	2	2	0
HR5	Other	5	5	1

Health Region	Facility type	Primary 2019 cohort	Strict balanced 2019–2024	Long balanced 2015–2024
HR5	PPI	14	14	13
HR5	Local Clinics	1	1	0
HR5	TOMY	16	16	0
HR6	Unknown	0	0	0
HR6	HC-HU	89	89	87
HR6	Mental HU	7	7	0
HR6	Other	1	1	0
HR6	PPI	20	19	18
HR6	Local Clinics	1	1	1
HR6	TOMY	32	32	0
HR7	HC-HU	17	17	15
HR7	Mental HU	3	3	0
HR7	Other	0	0	0
HR7	PPI	4	3	2
HR7	Local Clinics	1	1	1
HR7	TOMY	11	11	0
All regions	All types	581	543	378

Note: HC–HU combines Health Centres and Health Units for presentation; their administrative identifiers remain separate. Mental HU: mental health units; PPI: multipurpose regional clinics; TOMY: local health units. The strict balanced cohort is a subset of the primary cohort. The long-balanced cohort requires a non-missing count in all ten years and at least one positive count during 2015–2024. The flagged 2016 prescription-only value is excluded before eligibility is determined.

Table S2. Annual recorded activity and reporting coverage by Health Region, 2015–2024

HR1

Year	Regular	Emergency	Prescription-only	Other	Primary total
2015	2,038,572<br>56 / 103.0	751,834<br>54 / 88.9	424,999<br>47 / 114.6	188,782<br>49 / 117.9	3,215,405<br>100.6
2016	1,928,268<br>59 / 97.5	651,982<br>56 / 77.1	373,315<br>54 / 100.7	187,276<br>52 / 117.0	2,953,565<br>92.4
2017	2,209,196<br>55 / 111.7	744,649<br>54 / 88.0	437,321<br>53 / 117.9	235,138<br>51 / 146.9	3,391,166<br>106.1
2018	2,189,437<br>53 / 110.7	783,098<br>53 / 92.6	394,896<br>51 / 106.5	191,536<br>51 / 119.7	3,367,431<br>105.4
2019	1,978,338<br>65 / 100.0	846,104<br>65 / 100.0	370,860<br>64 / 100.0	160,075<br>63 / 100.0	3,195,302<br>100.0
2020	1,498,827<br>68 / 75.8	707,051<br>68 / 83.6	344,421<br>66 / 92.9	121,915<br>61 / 76.2	2,550,299<br>79.8
2021	1,398,792<br>68 / 70.7	699,842<br>68 / 82.7	280,381<br>65 / 75.6	28,625<br>62 / 17.9	2,379,015<br>74.5
2022	1,394,223<br>71 / 70.5	794,674<br>71 / 93.9	280,742<br>71 / 75.7	30<br>71 / 0.0	2,469,639<br>77.3
2023	1,430,929<br>69 / 72.3	854,782<br>69 / 101.0	303,605<br>69 / 81.9	1,559<br>64 / 1.0	2,589,316<br>81.0
2024	1,364,623<br>70 / 69.0	897,602<br>70 / 106.1	299,707<br>70 / 80.8	563<br>66 / 0.4	2,561,932<br>80.2

Note: For each visit category, the first line gives recorded visits; the second gives reporting facilities / activity index (2019 = 100). The primary-total column gives recorded visits and the activity index only. Reporting facilities have a non-missing count, including zero. Primary total sums regular, emergency and prescription-only visits. Indices use each region’s recorded 2019 total and describe the changing reporting network; they are not population rates or performance rankings. Other visits are shown descriptively only.

Table S2. Annual recorded activity and reporting coverage by Health Region, 2015–2024 (continued)

HR2

Year	Regular	Emergency	Prescription-only	Other	Primary total
2015	1,432,299<br>100 / 110.7	575,733<br>97 / 78.3	819,889<br>93 / 102.6	170,777<br>57 / 125.9	2,827,921<br>100.0
2016	1,407,885<br>107 / 108.8	593,526<br>107 / 80.7	914,337<br>107 / 114.4	175,047<br>96 / 129.0	2,915,748<br>103.1
2017	1,542,312<br>106 / 119.2	681,314<br>104 / 92.7	999,196<br>107 / 125.0	190,090<br>102 / 140.1	3,222,822<br>113.9
2018	1,550,567<br>107 / 119.8	733,444<br>104 / 99.7	923,973<br>106 / 115.6	168,423<br>100 / 124.2	3,207,984<br>113.4
2019	1,293,834<br>125 / 100.0	735,326<br>123 / 100.0	799,218<br>125 / 100.0	135,644<br>112 / 100.0	2,828,378<br>100.0
2020	1,079,103<br>128 / 83.4	526,123<br>126 / 71.5	738,623<br>128 / 92.4	65,206<br>118 / 48.1	2,343,849<br>82.9
2021	941,028<br>126 / 72.7	461,469<br>124 / 62.8	658,696<br>126 / 82.4	72,795<br>115 / 53.7	2,061,193<br>72.9
2022	1,061,259<br>126 / 82.0	561,809<br>126 / 76.4	710,238<br>126 / 88.9	77,793<br>126 / 57.4	2,333,306<br>82.5
2023	1,094,302<br>129 / 84.6	594,587<br>129 / 80.9	700,673<br>129 / 87.7	73,481<br>116 / 54.2	2,389,562<br>84.5
2024	1,024,713<br>131 / 79.2	575,243<br>128 / 78.2	625,812<br>131 / 78.3	66,256<br>117 / 48.8	2,225,768<br>78.7

Note: For each visit category, the first line gives recorded visits; the second gives reporting facilities / activity index (2019 = 100). The primary-total column gives recorded visits and the activity index only. Reporting facilities have a non-missing count, including zero. Primary total sums regular, emergency and prescription-only visits. Indices use each region's recorded 2019 total and describe the changing reporting network; they are not population rates or performance rankings. Other visits are shown descriptively only.

Table S2. Annual recorded activity and reporting coverage by Health Region, 2015–2024 (continued)

HR3

Year	Regular	Emergency	Prescription-only	Other	Primary total
2015	547,789<br>49 / 64.5	241,005<br>37 / 101.2	338,859<br>49 / 96.3	411,871<br>51 / 623.9	1,127,653<br>78.4
2016	861,938<br>55 / 101.5	197,568<br>51 / 82.9	368,455<br>56 / 104.8	61,967<br>51 / 93.9	1,427,961<br>99.2
2017	993,138<br>55 / 117.0	215,123<br>55 / 90.3	388,078<br>55 / 110.3	33,675<br>52 / 51.0	1,596,339<br>110.9
2018	889,555<br>56 / 104.8	233,546<br>55 / 98.0	352,366<br>55 / 100.2	27,861<br>51 / 42.2	1,475,467<br>102.5
2019	848,943<br>65 / 100.0	238,231<br>61 / 100.0	351,719<br>64 / 100.0	66,019<br>60 / 100.0	1,438,893<br>100.0
2020	659,031<br>75 / 77.6	149,910<br>72 / 62.9	424,828<br>74 / 120.8	58,909<br>71 / 89.2	1,233,769<br>85.7
2021	558,012<br>72 / 65.7	154,408<br>67 / 64.8	424,282<br>71 / 120.6	54,121<br>68 / 82.0	1,136,702<br>79.0
2022	665,155<br>74 / 78.4	206,720<br>74 / 86.8	391,262<br>74 / 111.2	55,262<br>74 / 83.7	1,263,137<br>87.8
2023	755,951<br>74 / 89.0	253,871<br>71 / 106.6	386,731<br>73 / 110.0	44,238<br>72 / 67.0	1,396,553<br>97.1
2024	749,938<br>74 / 88.3	271,991<br>71 / 114.2	393,352<br>73 / 111.8	42,764<br>72 / 64.8	1,415,281<br>98.4

Note: For each visit category, the first line gives recorded visits; the second gives reporting facilities / activity index (2019 = 100). The primary-total column gives recorded visits and the activity index only. Reporting facilities have a non-missing count, including zero. Primary total sums regular, emergency and prescription-only visits. Indices use each region's recorded 2019 total and describe the changing reporting network; they are not population rates or performance rankings. Other visits are shown descriptively only.

Table S2. Annual recorded activity and reporting coverage by Health Region, 2015–2024 (continued)

HR4

Year	Regular	Emergency	Prescription-only	Other	Primary total
2015	1,100,219<br>63 / 131.5	540,628<br>62 / 117.2	712,925<br>61 / 142.1	52,875<br>44 / 102.6	2,353,772<br>130.8
2016	994,498<br>70 / 118.8	453,423<br>68 / 98.3	682,824<br>70 / 136.1	46,721<br>64 / 90.7	2,130,745<br>118.4
2017	969,730<br>69 / 115.9	438,294<br>68 / 95.1	662,245<br>69 / 132.0	54,603<br>65 / 106.0	2,070,269<br>115.1
2018	857,549<br>68 / 102.5	435,981<br>65 / 94.6	510,349<br>68 / 101.8	51,160<br>62 / 99.3	1,803,879<br>100.2
2019	836,768<br>88 / 100.0	461,106<br>86 / 100.0	501,533<br>87 / 100.0	51,530<br>83 / 100.0	1,799,407<br>100.0
2020	542,566<br>94 / 64.8	332,534<br>92 / 72.1	554,709<br>92 / 110.6	63,453<br>88 / 123.1	1,429,809<br>79.5
2021	563,936<br>88 / 67.4	333,863<br>86 / 72.4	580,391<br>87 / 115.7	51,008<br>84 / 99.0	1,478,190<br>82.1
2022	471,796<br>87 / 56.4	431,960<br>87 / 93.7	520,780<br>87 / 103.8	89,137<br>87 / 173.0	1,424,536<br>79.2
2023	529,523<br>87 / 63.3	480,572<br>84 / 104.2	551,772<br>86 / 110.0	48,073<br>84 / 93.3	1,561,867<br>86.8
2024	611,659<br>89 / 73.1	503,632<br>87 / 109.2	554,464<br>87 / 110.6	48,682<br>85 / 94.5	1,669,755<br>92.8

Note: For each visit category, the first line gives recorded visits; the second gives reporting facilities / activity index (2019 = 100). The primary-total column gives recorded visits and the activity index only. Reporting facilities have a non-missing count, including zero. Primary total sums regular, emergency and prescription-only visits. Indices use each region’s recorded 2019 total and describe the changing reporting network; they are not population rates or performance rankings. Other visits are shown descriptively only.

Table S2. Annual recorded activity and reporting coverage by Health Region, 2015–2024 (continued)

HR5

Year	Regular	Emergency	Prescription-only	Other	Primary total
2015	560,822<br>58 / 80.9	236,786<br>56 / 74.2	500,311<br>57 / 80.3	62,695<br>31 / 115.6	1,297,919<br>79.3
2016	584,021<br>62 / 84.2	258,634<br>63 / 81.0	484,503<br>63 / 77.8	92,882<br>54 / 171.2	1,327,158<br>81.1
2017	673,348<br>62 / 97.1	319,213<br>62 / 100.0	558,285<br>62 / 89.6	95,420<br>61 / 175.9	1,550,846<br>94.8
2018	632,019<br>63 / 91.1	315,661<br>63 / 98.9	556,448<br>63 / 89.3	43,991<br>62 / 81.1	1,504,128<br>92.0
2019	693,541<br>82 / 100.0	319,160<br>80 / 100.0	623,088<br>81 / 100.0	54,254<br>75 / 100.0	1,635,789<br>100.0
2020	583,335<br>94 / 84.1	240,854<br>90 / 75.5	600,976<br>92 / 96.5	45,338<br>82 / 83.6	1,425,165<br>87.1
2021	550,009<br>87 / 79.3	253,238<br>87 / 79.3	586,367<br>87 / 94.1	43,894<br>81 / 80.9	1,389,614<br>85.0
2022	610,697<br>87 / 88.1	307,092<br>87 / 96.2	623,448<br>87 / 100.1	52,520<br>87 / 96.8	1,541,237<br>94.2
2023	638,484<br>89 / 92.1	357,608<br>89 / 112.0	662,581<br>89 / 106.3	53,357<br>86 / 98.3	1,658,673<br>101.4
2024	600,790<br>89 / 86.6	370,317<br>89 / 116.0	613,201<br>89 / 98.4	50,032<br>83 / 92.2	1,584,308<br>96.9

Note: For each visit category, the first line gives recorded visits; the second gives reporting facilities / activity index (2019 = 100). The primary-total column gives recorded visits and the activity index only. Reporting facilities have a non-missing count, including zero. Primary total sums regular, emergency and prescription-only visits. Indices use each region’s recorded 2019 total and describe the changing reporting network; they are not population rates or performance rankings. Other visits are shown descriptively only.

Table S2. Annual recorded activity and reporting coverage by Health Region, 2015–2024 (continued)

HR6

Year	Regular	Emergency	Prescription-only	Other	Primary total
2015	1,120,465<br>108 / 90.1	554,896<br>109 / 98.4	1,322,792<br>109 / 116.6	43,639<br>63 / 90.0	2,998,153<br>101.9
2016	1,098,569<br>121 / 88.3	481,647<br>120 / 85.4	1,209,789<br>119 / 106.6	25,958<br>111 / 53.5	2,790,005<br>94.8
2017	1,341,282<br>125 / 107.8	508,379<br>124 / 90.2	1,159,279<br>125 / 102.2	47,254<br>118 / 97.4	3,008,940<br>102.3
2018	1,251,718<br>123 / 100.6	529,327<br>121 / 93.9	1,093,438<br>123 / 96.4	36,834<br>112 / 75.9	2,874,483<br>97.7
2019	1,243,996<br>156 / 100.0	563,875<br>154 / 100.0	1,134,708<br>156 / 100.0	48,502<br>142 / 100.0	2,942,579<br>100.0
2020	970,284<br>156 / 78.0	454,549<br>154 / 80.6	1,052,639<br>156 / 92.8	37,293<br>143 / 76.9	2,477,472<br>84.2
2021	1,003,447<br>156 / 80.7	459,484<br>155 / 81.5	1,037,834<br>156 / 91.5	45,358<br>146 / 93.5	2,500,765<br>85.0
2022	1,043,284<br>155 / 83.9	539,544<br>155 / 95.7	1,076,175<br>155 / 94.8	47,583<br>155 / 98.1	2,659,003<br>90.4
2023	1,129,065<br>158 / 90.8	571,901<br>157 / 101.4	1,135,432<br>158 / 100.1	40,979<br>145 / 84.5	2,836,398<br>96.4
2024	1,049,874<br>158 / 84.4	600,860<br>157 / 106.6	1,168,632<br>158 / 103.0	38,832<br>143 / 80.1	2,819,366<br>95.8

Note: For each visit category, the first line gives recorded visits; the second gives reporting facilities / activity index (2019 = 100). The primary-total column gives recorded visits and the activity index only. Reporting facilities have a non-missing count, including zero. Primary total sums regular, emergency and prescription-only visits. Indices use each region's recorded 2019 total and describe the changing reporting network; they are not population rates or performance rankings. Other visits are shown descriptively only.

Table S2. Annual recorded activity and reporting coverage by Health Region, 2015–2024 (continued)

HR7

Year	Regular	Emergency	Prescription-only	Other	Primary total
2015	369,717<br>20 / 74.5	165,290<br>19 / 75.9	294,783<br>20 / 107.2	87,780<br>16 / 103.3	829,790<br>83.9
2016	355,459<br>27 / 71.6	161,149<br>25 / 74.0	277,881<br>26 / 101.0	82,088<br>24 / 96.6	794,489<br>80.3
2017	407,050<br>25 / 82.0	162,357<br>25 / 74.6	289,950<br>25 / 105.4	96,683<br>24 / 113.8	859,357<br>86.9
2018	416,813<br>27 / 83.9	166,019<br>27 / 76.2	252,302<br>27 / 91.7	89,686<br>27 / 105.6	835,134<br>84.4
2019	496,542<br>39 / 100.0	217,752<br>39 / 100.0	274,996<br>39 / 100.0	84,954<br>38 / 100.0	989,290<br>100.0
2020	415,639<br>41 / 83.7	165,664<br>41 / 76.1	262,173<br>41 / 95.3	65,503<br>39 / 77.1	843,476<br>85.3
2021	434,985<br>42 / 87.6	168,429<br>42 / 77.3	256,676<br>42 / 93.3	66,974<br>41 / 78.8	860,090<br>86.9
2022	419,256<br>42 / 84.4	176,221<br>42 / 80.9	250,747<br>42 / 91.2	56,995<br>42 / 67.1	846,224<br>85.5
2023	435,273<br>41 / 87.7	192,034<br>41 / 88.2	259,777<br>41 / 94.5	26,859<br>39 / 31.6	887,084<br>89.7
2024	423,871<br>42 / 85.4	164,365<br>42 / 75.5	245,570<br>42 / 89.3	37,393<br>40 / 44.0	833,806<br>84.3

Note: For each visit category, the first line gives recorded visits; the second gives reporting facilities / activity index (2019 = 100). The primary-total column gives recorded visits and the activity index only. Reporting facilities have a non-missing count, including zero. Primary total sums regular, emergency and prescription-only visits. Indices use each region’s recorded 2019 total and describe the changing reporting network; they are not population rates or performance rankings. Other visits are shown descriptively only.

Table S3. Full year-specific estimates in the long-balanced 2015–2024 cohorts

Regular visits: 394 facilities and 3,940 observations

Year	IRR	95% CI	p value	Difference from 2019 (%)
2015	1.079	1.005–1.158	0.036	7.9
2016	1.081	1.014–1.152	0.017	8.1
2017	1.211	1.154–1.271	<0.001	21.1
2018	1.148	1.107–1.191	<0.001	14.8
2019	1.000 (reference)	—	—	0.0
2020	0.752	0.727–0.777	<0.001	-24.8
2021	0.723	0.687–0.762	<0.001	-27.7
2022	0.760	0.723–0.798	<0.001	-24.0
2023	0.796	0.754–0.840	<0.001	-20.4
2024	0.763	0.723–0.805	<0.001	-23.7

Note: Conditional facility fixed-effects Poisson models with facility-clustered standard errors and 2019 as reference. IRRs describe expected recorded annual counts relative to 2019; they are not causal pandemic effects. Confidence intervals and p values are pointwise. Each facility has a non-missing count in all ten years and at least one positive count during 2015–2024. The flagged 2016 prescription-only value is excluded before cohort eligibility is determined.

Table S3. Full year-specific estimates in the long-balanced 2015–2024 cohorts (continued)

Emergency visits: 369 facilities and 3,690 observations

Year	IRR	95% CI	p value	Difference from 2019 (%)
2015	0.952	0.886–1.022	0.171	-4.8
2016	0.873	0.817–0.933	<0.001	-12.7
2017	0.963	0.915–1.013	0.140	-3.7
2018	1.003	0.971–1.035	0.867	0.3
2019	1.000 (reference)	—	—	0.0
2020	0.731	0.705–0.759	<0.001	-26.9
2021	0.722	0.683–0.762	<0.001	-27.8
2022	0.887	0.832–0.946	<0.001	-11.3
2023	0.964	0.907–1.023	0.226	-3.6
2024	0.972	0.907–1.040	0.408	-2.8

Note: Conditional facility fixed-effects Poisson models with facility-clustered standard errors and 2019 as reference. IRRs describe expected recorded annual counts relative to 2019; they are not causal pandemic effects. Confidence intervals and p values are pointwise. Each facility has a non-missing count in all ten years and at least one positive count during 2015–2024. The flagged 2016 prescription-only value is excluded before cohort eligibility is determined.

Table S3. Full year-specific estimates in the long-balanced 2015–2024 cohorts (continued)

Prescription-only visits: 378 facilities and 3,780 observations

Year	IRR	95% CI	p value	Difference from 2019 (%)
2015	1.222	1.124–1.328	<0.001	22.2
2016	1.173	1.079–1.274	<0.001	17.3
2017	1.197	1.131–1.267	<0.001	19.7
2018	1.094	1.055–1.135	<0.001	9.4
2019	1.000 (reference)	—	—	0.0
2020	0.955	0.916–0.995	0.028	-4.5
2021	0.921	0.868–0.976	0.005	-7.9
2022	0.926	0.875–0.979	0.007	-7.4
2023	0.956	0.896–1.021	0.177	-4.4
2024	0.927	0.855–1.005	0.067	-7.3

Note: Conditional facility fixed-effects Poisson models with facility-clustered standard errors and 2019 as reference. IRRs describe expected recorded annual counts relative to 2019; they are not causal pandemic effects. Confidence intervals and p values are pointwise. Each facility has a non-missing count in all ten years and at least one positive count during 2015–2024. The flagged 2016 prescription-only value is excluded before cohort eligibility is determined.

Table S4. Long-balanced prescription-only sensitivity retaining the extreme 2016 value

Prescription-only visits: 379 facilities and 3,790 observations

Year	IRR	95% CI	p value	Difference from 2019 (%)
2015	1.218	1.122–1.323	<0.001	21.8
2016	9.879	1.777–54.925	0.009	887.9
2017	1.192	1.127–1.262	<0.001	19.2
2018	1.097	1.057–1.138	<0.001	9.7
2019	1.000 (reference)	—	—	0.0
2020	0.950	0.911–0.991	0.017	-5.0
2021	0.919	0.868–0.974	0.004	-8.1
2022	0.926	0.875–0.979	0.007	-7.4
2023	0.956	0.896–1.020	0.174	-4.4
2024	0.929	0.857–1.006	0.070	-7.1

Note: The flagged count of 28,132,398 prescription-only visits in 2016 is retained, and cohort eligibility is re-derived. This cohort includes 379 facilities, compared with 378 in Table S3; the sensitivity therefore also changes cohort membership. The cause of the flagged value is unverified. Conditional facility fixed-effects Poisson models with facility-clustered standard errors and 2019 as reference. IRRs describe expected recorded annual counts relative to 2019; they are not causal pandemic effects. Confidence intervals and p values are pointwise.

Table S5. Joint Wald tests of the 2015–2018 annual level contrasts relative to 2019

Outcome	Wald χ^2	df	p value	Facilities	Observations
Regular visits	112.547	4	<0.001	394	3,940
Emergency visits	32.627	4	<0.001	369	3,690
Prescription-only visits	55.949	4	<0.001	378	3,780

Note: Tests use the long-balanced models in Table S3 and facility-clustered covariance matrices. The null hypothesis is that the 2015–2018 year coefficients jointly equal zero relative to 2019. Rejection indicates differences in annual levels, not a significant linear trend. These analyses do not provide a no-pandemic counterfactual.
